# Acetabulum Protrusion Following Ceramic Liner Fracture: A Case Report

**DOI:** 10.5704/MOJ.1803.010

**Published:** 2018-03

**Authors:** K Aytekin, CZ Esenyel

**Affiliations:** Department of Orthopaedics and Traumatology, University of Giresun, Giresun, Turkey

**Keywords:** ceramic liner, ceramic head, protrusio acetabuli, ceramic fracture, revision arthroplasty

## Abstract

Hip arthroplasty is an extremely satisfying treatment method for coxarthrosis which is in increasing use throughout the world. However, loosening of the prosthesis is a significant complication and to overcome this, ceramic liners are increasingly being selected. If the survival of ceramic surfaces is prolonged, there is a risk of fracture of the ceramic materials. New ceramic materials developed to overcome this problem are more resistant. The case presented here is of a patient in whom liner fracture developed following ceramic-ceramic hip arthroplasty. The ceramic femoral head was observed to have protruded into the defect created in the acetabular component. Acetabular revision was applied to the patient.

## Introduction

Hip arthroplasty is an extremely satisfying treatment method for coxarthrosis. However, with the possibility of loosening following arthroplasty, one potential problem to be overcome is that isolated acetabular loosening may be observed following hip arthroplasty.

One of the most common reasons for failure is aseptic loosening. Throughout the world, polyethylene liners are used most in hip arthroplasty but because of delayed osteolysis and loosening in the component, ceramic materials have come into use^1^. The main problem with ceramic material is its fragility^1^. In the current report, a case is presented where a ceramic femoral head created a defect in the acetabular component as a result of breakage of the ceramic liner following hip arthroplasty using ceramic head and ceramic insert. This report can be considered of value as it is uncommon for a defect to have been created in the acetabular component and for the ceramic head continue to survive without fracture.

## Case Report

**Fig. 1:**
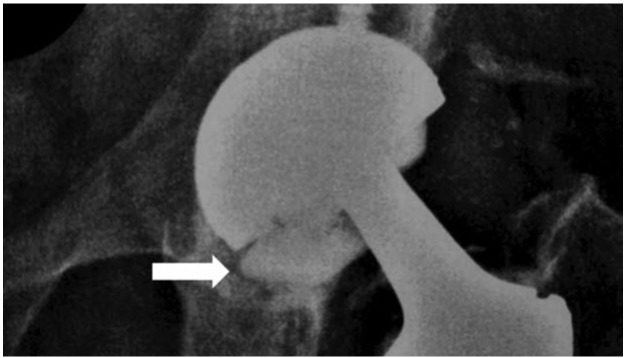
Preoperative radiograph (fractured ceramic liner indicated by arrow).

A 57-year old female presented at the Orthopaedics and Traumatology polyclinic with complaints of pain in the left hip. A prosthesis had been applied to the left hip six years previously in another hospital. Pain which had started very mildly 4-5 years previously had increased over time. Because the surgery was performed in another center, we were unable to ascertain if there was any problem during the surgery or inserting the ceramic liner, and there was no recollection of any trauma in the patient’s anamnesis. It was later ascertained that Smith and Nephew EP-FIT plus ceramic insert was used in the first surgery. The patient had no complaints of any abnormal sound arising from the hip. The findings of the physical and radiological examinations were consistent with aseptic loosening. On the hip radiograph, there was noted asymmetry in the centralisation of the femoral head within the acetabulum (Fig. 1). Revision surgery was planned for loosening of acetabular component. The patient was operated on using the previous incision site. There was no loosening of the femoral component. The femoral head was ceramic. The ceramic liner was totally fragmanted and detached from the acetabular component, which was observed to be in approximately 10 degrees retroversion. The pieces of the ceramic liner were as small as seen in the radiograph (Fig. 1).

**Fig. 2:**
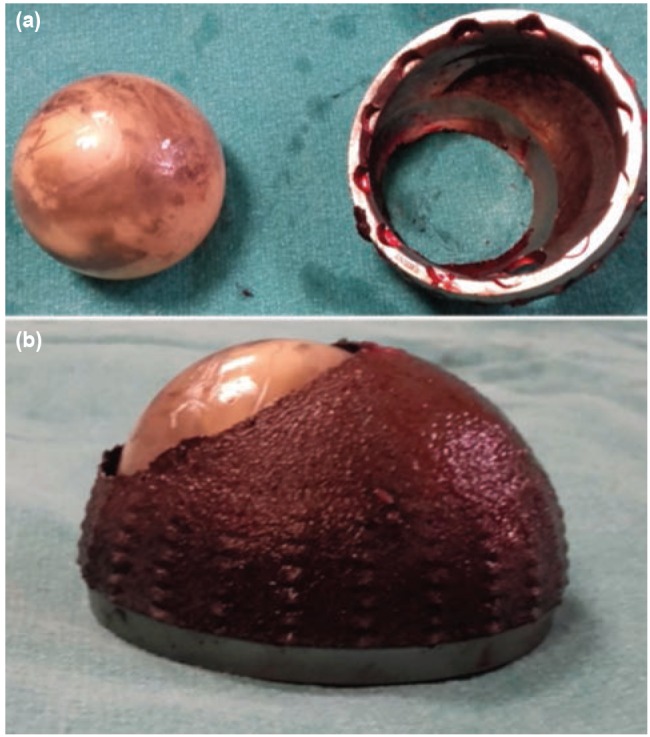
Ceramic femoral head and acetabular cup where the defect had formed, removed from the patient. The surfaces of the ceramic head and the acetabular component contacting each other (a) and contacting to bone (b).

**Fig. 3:**
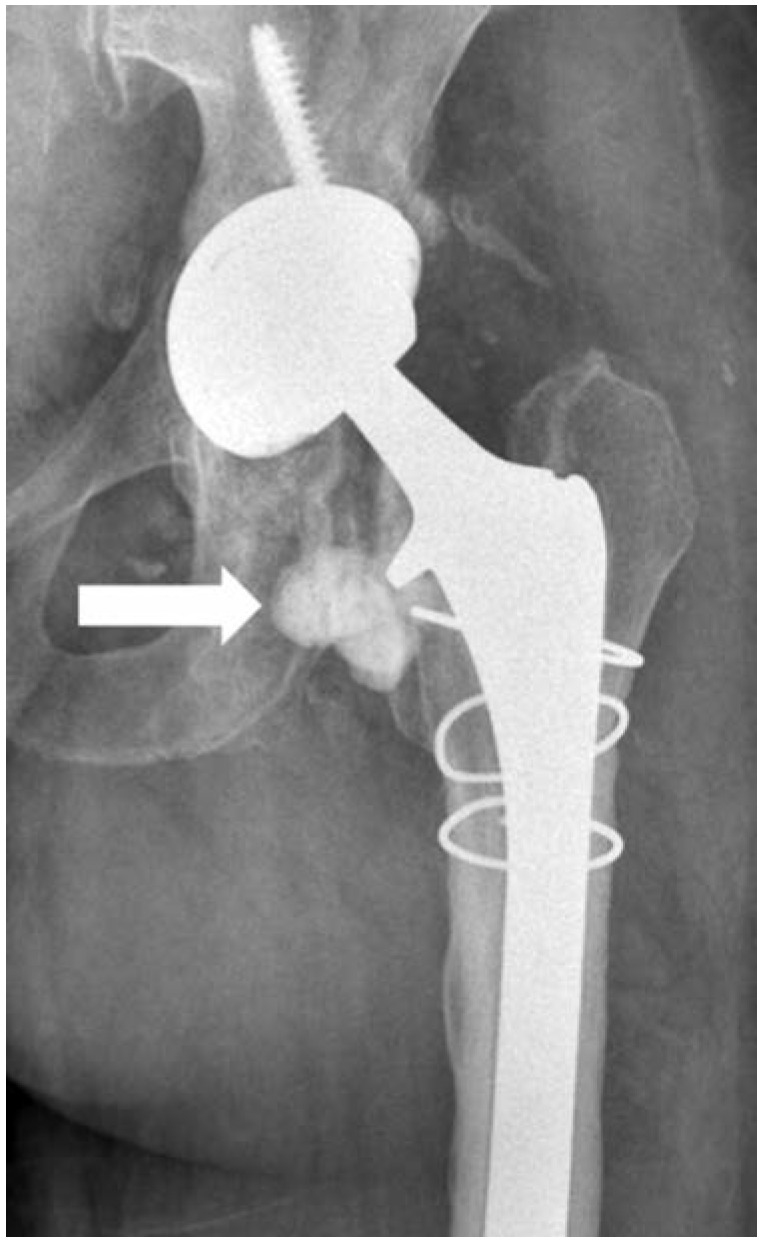
Radiograph after revision (fractured ceramic liner away from joint indicated by arrow).

There was widespread debris in the joint. A defect, approximately 28mm in diameter, was observed in the weight-bearing area in the centre of the acetabular component (Fig. 2). This defect was thought to have been formed by the ceramic femoral head settling in this area. The acetabular component and one screw were removed. The head of the second screw was observed to have been completely smoothed out and not causing any damage to the bone stock and was not removed. The acetabulum was then reamed and grafted and the acetabular cup was fixed with two screws in appropriate anteversion. It was observed that the ceramic liner pieces remaining around the joint did not lead to restriction of the hip movements after the trial components were placed, and it was decided not to excise these ceramic liner pieces (Fig. 3, indicated by arrow). The revision surgery was completed using a ceramic insert and ceramic head (Fig. 3). The patient did not have any complaint about her hip in the post-operative two years follow-up.

## Discussion

Hip arthroplasty is being applied at increasing frequency worldwide. Various materials are used to prolong the survival of the components in parallel with increasing human life expectancy^2,3^. In hip arthroplasty applications throughout the world, there is an increasing frequency of selection of ceramic head and ceramic liner as osteolysis occurs at a lower rate and at a later stage^1^. Fracture is one of the most commonly observed complications in patients where ceramic materials are used^2^. However, new ceramic materials are more resistant^1^. Theoretically, the advantages of a ceramic head are that it is smoother, harder and more slippery. Therefore, there is less friction between the two weight-bearing surfaces, more lubrication and less scratching, thereby providing longer survival of the ceramic head^2^. The importance of the positioning of the components in hip prosthesis for the survival of the prosthesis is well known^4^.

Malalignment in the acetabular cup or fixing the ceramic liner in the wrong position can cause fracture of the ceramic liner^1^. Fractures of the ceramic head and ceramic liner have been reported in the literature. Malposition of cup and misalingment of ceramic liner are the two underlying reasons for liner fractures^1^. Ceramic liner fractures are not directly related to trauma^1^ as in this case report. A possible cause may be impingiment of the femoral neck of the stem and acetabular component^1^. In reports, most patients with ceramic liner fracture had complained of noise before the liner fracture developed^1^. But our patient gave no history of complaints at all of noise before the fracture developed. Radiographs taken after ceramic head fracture have previously shown an asymmetric position of the femoral head within the acetabular component and the radiographic imge of the ceramic liner^1^. In the case presented here, asymmetry of the femoral head within the acetabular component and parts of the fractured ceramic liner were clearly observed in the radiographs (Fig. 1, indicated by arrow). After fracture of the ceramic liner, revision is necessary for the defect seen in the acetabular component^3^.

In the current case, following the development of the ceramic liner fracture in the base of acetabular cup which was placed in a retrovert position, the ceramic head continued without breakage but a defect developed in the load-bearing area where the screw holes were (Fig. 2). It is known that titanium acetabular cup is smoother than ceramic head. The probable reason for this defect is that the new ceramic materials are more resistant. Steinhoff *et al* published intraoperative findings of ceramic liner fracture^4^. In all cases there was one or more symptoms related to malpositon of component; squeaking or impingiment. Hasegawa *et al* reported a case with fracture of modular acetabular component associated neck impingiment^5^. As we emphasized before, the most unique occurance in our case was the formation of a defect at the acetabular cup after fracture of the ceramic liner.

Our patient had no complaints of any hip pain at two-year follow-up.

In conclusion, ceramic materials are strong and resistant, and together with appropriate component alignment in total hip arthroplasty ceramic head and ceramic liner can be considered to prolong prosthesis survival. The possibility of liner fracture should be kept in mind in patients where a ceramic liner had been used.

## Conflict of Interest

The authors declare no conflicts of interest.

## References

[1] Traina F, De Fine M, Di Martino A, Faldini C. (2013). Fracture of ceramic bearing surfaces following total hip replacement: a systematic review. Biomed Res Int..

[2] Cafri G, Paxton EW, Love R, Bini SA, Kurtz SM (2017). Is there a difference in revision risk between metal and ceramic heads on highly crosslinked polyethylene liners?. Clin Orthop Relat Res..

[3] Si HB, Zeng Y, Cao F, Pei FX, Shen B (2015). Is a ceramic-on-ceramic bearing really superior to ceramic-on-polyethylene for primary total hip arthroplasty? A systematic review and meta-analysis of randomised controlled trials. Hip Int..

[4] Steinhoff A, Hakim V, Walker RH, Colwell CW, Copp SN (2015). Ceramic liner fracture and impingement in total hip arthroplasty. HSS J.

[5] Hasegawa M, Sudo A, Hirata H, Uchida A (2003). Ceramic acetabular liner fracture in total hip arthroplasty with a ceramic sandwich cup. J Arthroplasty..

